# Seed Priming with Salicylic Acid Alleviates Salt Stress Toxicity in Barley by Suppressing ROS Accumulation and Improving Antioxidant Defense Systems, Compared to Halo- and Gibberellin Priming

**DOI:** 10.3390/antiox12091779

**Published:** 2023-09-18

**Authors:** Hasna Ellouzi, Walid Zorrig, Souhir Amraoui, Samia Oueslati, Chedly Abdelly, Mokded Rabhi, Kadambot H. M. Siddique, Kamel Hessini

**Affiliations:** 1 Laboratory of Extremophile Plants, Centre of Biotechnology of Borj-Cedria (CBBC), BP901, Hammam-Lif 2050, Tunisia; ellouzihasnaa@gmail.com (H.E.); walid.zorrig@cbbc.rnrt.tn (W.Z.); souhir2016.amraoui@gmail.com (S.A.); oueslatisamia01@gmail.com (S.O.); abdelly.chedly@gmail.com (C.A.); 2 Department of Plant Production and Protection, College of Agriculture and Veterinary Medicine, Qassim University, Buraydah 51452, Saudi Arabia; mokded.rabhi@gmail.com; 3 The UWA Institute of Agriculture, The University of Western Australia, Perth, WA 6001, Australia; kadambot.siddique@uwa.edu.au; 4 Department of Biology, College of Sciences, Taif University, P.O. Box 11099, Taif 21944, Saudi Arabia

**Keywords:** *Hordeum vulgare*, hormo-priming, redox homeostasis, salicylic acid, salt stress, stress memory

## Abstract

Plants are highly sensitive to various environmental stresses, which can hinder their growth and reduce yields. In this study, we investigated the potential of seed priming with salicylic acid (SA), gibberellic acid (GA_3_), and sodium chloride (NaCl) to mitigate the adverse effects of salinity stress in *Hordeum vulgare* at the germination and early seedling stages. Exposing *H. vulgare* seeds to salt stress reduced the final germination percentage and seedling shoot and root growth. Interestingly, all seed treatments significantly improved salt-induced responses, with GA_3_ being more effective in terms of germination performance, plant growth, and photosynthesis. SA priming exhibited promising effects on antioxidant defense mechanisms, proline, sugar, and ascorbic acid production. Notably, SA priming also suppressed reactive oxygen species accumulation and prevented lipid peroxidation. These findings highlight the ability of SA to manage crosstalk within the seed, coordinating many regulatory processes to support plant adaptation to salinity stress.

## 1. Introduction

Environmental stressors significantly challenge global food security, impacting crop yields and productivity. Among these stressors, salt stress is one of the most severe, affecting at least 20% of crop production [1]. Salt stress disrupts plant growth by disturbing osmotic and ionic homeostasis, affecting photosynthesis, generating reactive oxygen species (ROS), interfering with phytohormonal functions, and altering metabolic pathways and gene expression patterns [2].

Seed germination and early seedling growth are the most salt-sensitive plant growth stages. Salinity can delay seed germination by reducing water availability and altering the mobilization of stored reserves [3]. Therefore, improving plant tolerance to salt stress requires improving germination performance under saline conditions. The establishment of ‘vigorous crops’ under stressful conditions depends on successful germination [4]. However, germination is a heterogeneous biological process, with variations in timings and patterns among seeds. While strategies such as genetic engineering and conventional breeding have been used to mitigate the adverse effects of salinity, they have limitations in terms of success, biosafety restrictions, cost, and time [5,6]. Therefore, there is a critical need for simple, effective, low-cost, and low-risk methods to minimize the impact of salinity. Seed priming has emerged as a potential alternative approach that leverages plant stress memory and immune system activation to promote seed germination and enhance plant growth under various abiotic stresses without the need for genetic engineering [7]. Furthermore, immune stimulation through priming may confer improved defensive capabilities, which can be inherited epigenetically across plant generations [8], emphasizing the importance of understanding priming mechanisms.

Priming, also known as hardening, can occur naturally or be induced through exposure to specific agents [9]. Different types of seed priming include hydropriming, osmopriming, nutripriming, redox priming, and hormopriming [10]. Generally, seed priming treatments enhance germination performance, early seedling growth, and mineral and water uptake under various abiotic stresses, such as heat [11], drought [12], and salinity [13]. However, the specific effects of priming are influenced by factors such as priming duration [14] (Dai et al., 2017), priming agent concentration [13], and mode of action [15]. Priming acts as a signaling mechanism that triggers specific stress response pathways in primed seeds, enabling plants to respond more quickly and efficiently when subsequently exposed to environmental stresses, a phenomenon known as memory or a primed state [9,15].

Various chemical compounds, whether natural or synthetic, can induce a primed state in plants, enhancing their ability to tolerate salt stress [9]. These compounds include ROS such as H_2_O_2_ [15], H_2_S [16], melatonin [17], NO [18], silicon [13], and vitamins such as ascorbic acid [19], which are ideal priming agents for stimulating stress memory, so that seeds may overcome stresses during germination and prepare plants to better defend against external factors [20].

Phytohormones such as auxins (IAAs), cytokinins (CKs), gibberellins (GAs), ethylene (ET), abscisic acid (ABA), salicylic acid (SA), brassinosteroids (BRs), and jasmonates (JAs) play important roles in plant metabolism and development [21,22]. They also function as paramount chemical messengers, control several cellular processes in plants under normal and stressful conditions [23], and interact with each other, forming a signaling network from seed germination to maturation [24]. Recent research highlighted the significance of phytohormones as priming agents in mitigating abiotic stresses such as salt stress [23] and improving salt tolerance in cereal food crops [25].

Phytohormones interact with other metabolites, such as ROS, in the signaling cascade that regulates plant responses during priming [26]. For example, priming maize seeds with 28-homobrassinolide enhanced antioxidant enzyme activities, minimizing lipid peroxidation in maize seedlings grown under salt stress [27]. Seed priming with spermidine promoted salt tolerance in rice plants by reducing ROS accumulation and triggering the expression of genes encoding antioxidant enzymes. Moreover, seed priming or foliar application with JA mitigates salt stress in many plant species by scavenging ROS [28]. GA is also widely used in priming as a key mediator between the perception of an environmental signal and growth response [29]. In this context, soaking wheat grains with GA_3_ alleviated nutritional disorders caused by salinity better than ABA priming [30]. In another study, pre-treating wheat seeds with GA_3_ significantly decreased Na^+^ content but increased the activities of two key enzymes involved in amino acid biosynthesis (arginine decarboxylase and ornithine decarboxylase) under salt stress [31]. Similarly, seed priming with GA_3_ enhanced ion uptake, photosynthesis, and redox homeostasis in *Vicia faba* species cultivated under saline conditions [32]. We also showed the effective role of GA_3_ seed priming in cauliflower seedlings exposed to severe salinity [15]. Likewise, priming summer squash seeds simultaneously with GA_3_ and JA increased the production of catalase (CAT), peroxidase (APX), and superoxide dismutase (SOD) as an adaptive mechanism to salt stress [33]. Moreover, pre-treating corn seeds with SA increased α-amylase and leaf antioxidant enzyme activities, indicating that SA may contribute to establishing a redox balance by stimulating the antioxidant defense system [34].

Plants accumulate many non-toxic compounds under salt stress, such as sugars, proline, and glycine-betaine (GB) that act as osmoprotectants, helping them withstand osmotic stress without hindering regular metabolism [35]. Some studies have suggested a regulatory mechanism linking osmolytes and phytohormones in plant responses to salinity [35,36]. For example, foliar application of GA_3_ stimulated proline accumulation in salt-stressed *Linum usitatissimum* plants [37]. In contrast, a GA_3_ treatment decreased the proline content in *Zea mays* seedlings grown under saline conditions [38]. JA and BRs also modulate osmolyte production under stressful conditions [36]. Also, SA treatment mitigated salt stress toxicity in maize via sugar and proline accumulation [39]. Likewise, SA alleviated salinity stress in *Vigna radiata* seedlings in association with GB accumulation [40].

Therefore, this study investigates the effects of seed soaking with different priming agents (NaCl, SA, and GA_3_) on seed germination, seedling growth, ion homeostasis, and osmoprotectant production in barley (*Hordeum vulgare*) plants grown under saline and non-saline conditions. This study also focuses on dealing with osmotic and oxidative stress induced by salinity.

## 2. Materials and Methods

### 2.1. Priming Treatment and Growth Conditions

**Figure 1:**
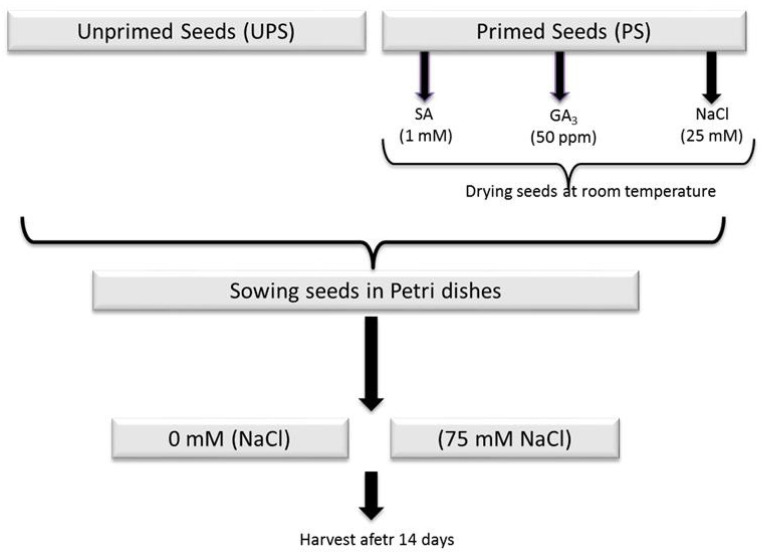
Experimental design. Barley seeds were soaked for 12 h in salicylic acid (10 mM) or gibberellic acid (50 ppm) solution. After drying to their original moisture content, primed and unprimed seeds were germinated in Petri dishes in the dark at 22 °C and watered every two days with distilled water (control) or 75 mM NaCl for 6 days. The Petri dishes were transferred to a growth chamber with continuous light illumination (100 μmol photons m^−2^ s^−1^) at 22 °C for 14 days before the seedlings were harvested.

Barley seeds (Manel variety) were disinfected with a diluted sodium hypochlorite (NaOCl) solution (50 µL of NaOCl in 150 mL of distilled water) for 5 min and washed three times with distilled water. The seeds were soaked in salicylic acid (SA; 1 mM), gibberellic acid (GA_3_; 50 ppm), or NaCl (25 mM) solution for 12 h at 25 °C. Afterward, the seeds were oven-dried at 25 °C to the initial weight of unprimed seeds for 48 h. Figure 1 illustrates the experimental design.

Primed (P) and unprimed (UP) seeds were germinated in Petri dishes on moistened double layers of filter paper, with 20 seeds per dish. The Petri dishes were kept in the dark at 22 °C and irrigated every two days with distilled water (control) or 75 mM NaCl for 6 days. The Petri dishes were arranged in a completely randomized design with three replicates per treatment and 20 seeds per Petri dish. Subsequently, the Petri dishes were maintained under a 16 h light/8 h dark regime in the same constant environment chamber set at 22 °C for two weeks, at which time the seedlings were harvested and analyzed. It is important to mention that all data are the means of four replicates for the overall analyzed parameters.

### 2.2. Germination Traits

The germination dynamics of P and UP seeds of *H. vulgare* sown under control and saline conditions were evaluated by calculating mean germination time (MGT) and final germination percentage (FGP) using the method described by Al-Mudaris [41]:

MGT = ΣF × X/ΣF (1)

where F is the number of seeds germinated on day X.

FGP = (the total number of seeds germinated at the end of the trial × 100)/(the number of initial seeds used).

### 2.3. Relative Water Content (RWC) Measurement

Seedlings were harvested and separated into shoots and roots, and their fresh weights (FW) were recorded. The shoot and root samples were then incubated in 30 mL of distilled water in the dark for 24 h to determine the turgid weight (TW). Dry weights (DWs) of both organs were obtained by oven-drying the samples at 60 °C. RWC was calculated as per Sairam et al. [42]:

RWC = (FW − DW) × 100/(TW − DW)(2)

### 2.4. Total Chlorophyll Content

Total chlorophyll content was measured using the method described by Lichtenthaler [43]. Briefly, 100 mg of fresh shoot samples were homogenized in 5 mL of 80% acetone and incubated in the dark at 4 °C for 72 h, before measuring the absorbance of the extract using a UV-visible spectrophotometer (Dual Beam 8 Auto Cell UVS-2700) at 470, 646, and 663 nm wavelengths.

### 2.5. Total Sugar Content

Soluble sugars in shoots and roots were measured using anthrone reagent, as described in [44] Yemm and Willis.

### 2.6. Sodium (Na^+^) and Potassium (K^+^) Analysis

For ion extraction, shoot and root dry matter was incubated in H_2_SO_4_ (1 N) at 80 °C for 1 h [45] before measuring Na^+^ and K^+^ contents using flame photometry (BWB flame photometer, BWB Technologies, Newbury, UK).

### 2.7. Hydrogen Peroxide (H_2_O_2_) Content

H_2_O_2_ content was determined according to the method of Sergiev et al. [46]. Fresh shoot and root tissues were homogenized in 5% (*w*/*v*) TCA before centrifuging the homogenate at 10,000× *g* for 15 min at 4 °C. The supernatant was mixed with 10 mM potassium phosphate buffer (pH 7.0) and 1 M KI before measuring the absorbance at 390 nm. The H_2_O_2_ content was calculated using a standard calibration curve.

### 2.8. Lipid Peroxidation (MDA) Content

The degree of lipid peroxidation was assayed by determining the malondialdehyde (MDA) content (nmol/g FW) in shoots and roots, as described by Hodge et al. [47].

### 2.9. Proline Accumulation

Proline content was assayed according to Bates et al. [48]. For proline extraction, fresh shoot and root samples were homogenized in 2 mL of ethanol, with the mixture heated at 85 °C in a water bath for 1 h. The reaction was stopped by placing the tubes in an ice bath. Subsequently, a solution was prepared comprising 1 mL of the upper phase of the tube mixed with 1 mL of acetic acid and 1 mL of a mixture containing 120 mL of distilled water, 300 mL of acetic acid, 300 mL of orthophosphoric acid, and 25 mg of ninhydrin. The solution was boiled for 30 min. After cooling, 5 mL of toluene was added to the mixture. The proline phase was collected and the absorbance was read at 528 nm. Proline content was calculated in ng/g FW.

### 2.10. Ascorbic Acid (AsA) Content

Total ascorbic acid (AsA) content was determined in shoot and root samples using the method in Kampfenkel et al. [49].

### 2.11. Polyphenol Quantification

Phenolic compounds were determined using the Folin–Ciocalteu reagent [50]. Briefly, dry leaf samples were extracted using pure methanol for 30 min, with the extract kept at 4 °C for 24 h. An aliquot of the extract was added to 125 µL of Folin–Ciocalteu reagent and 500 µL of deionized water, shaken, and incubated for 6 min before adding 1.25 mL of 7% Na_2_CO_3_ solution. The solution was diluted with deionized water to a final volume of 3 mL. After that, the reaction mixture was incubated at 23 °C for 90 min, with the absorbance measured at 760 nm. The total phenolic content in the shoots is expressed as mg gallic acid equivalents (GAE) per gram of dry weight (mg GAE/g DW).

### 2.12. Antioxidant Enzyme Extraction and Assay

Antioxidant enzymes were extracted from frozen shoot and root samples using polyvinyl pyrrolidone in 50 mM K-phosphate buffer (pH 7.8) containing 10 mM ethylenediaminetetra-acetic acid (EDTA), 1 mM dithiothreitol, and 0.1 mM phenylmethylsulfonyl fluoride (PMSF). After centrifugation at 12,000× *g* for 30 min, the supernatant was collected and used for different enzyme assays. Total protein content was determined using the Bradford method [51], with bovine serum albumin as the standard.

Total superoxide dismutase (SOD, EC 1.11.1.5) activity was assayed according to [52] (Scebba et al., 1999). Total catalase (CAT, EC 1.11.1.6) activity was measured by monitoring the decrease in absorbance at 240 nm using spectrophotometry [53]. Guaiacol peroxidase (GPX, EC 1.11.1.7) activity was determined by monitoring the increase in absorbance at 470 nm, following the method described by Fielding and Hall [54].

For phenylalanine ammonia-lyase (PAL) and tyrosine ammonia-lyase (TAL) activities, shoot samples (100 mg) were extracted in 50 mM Tris-HCl buffer (pH 8.0) containing 14.4 mM 2-mercaptoethanol and 1% (*w*/*v*) polyvinyl polypyrrolidone (PVP). The homogenates were centrifuged at 15,000× *g* for 10 min at 4 °C and assayed using the method of Berner et al. [55]. For PAL activity, the reaction mixture contained 50 mM Tris-HCl buffer (pH 8.0) and the enzyme extract, with the reaction initiated by adding 15 mM L-phenylalanine. The mixture was incubated for 70 min at 37 °C, with the reaction stopped by adding HCl (5N). The amount of trans-cinnamic acid formed was determined by measuring the absorbance at 290 nm. A molar extinction coefficient of 17.4 mM cm^–1^ was used to quantify the cinnamic acids formed during the enzymatic reaction. For TAL activity, the reaction medium comprised 150 mM L-tyrosine and 3 mL of the extraction buffer. The mixture was incubated at 30 °C for 30 min, with TAL activity measured by monitoring the formation of hydroxycinnamic acid at 310 nm.

### 2.13. Statistical Analysis

Statistical analysis was conducted using the software package SPSS version 21.0 (SPSS Inc., Chicago, USA). Differences between priming treatments at a given salinity level were determined using one-way analysis of variance (ANOVA) according to Duncan’s multiple range tests (*p* ≤ 0.05). Comparisons between 0 and 75 mM NaCl for a given priming treatment was performed according to Student’s *t*-test 6 (*p* ≤ 0.05).

## 3. Results

### 3.1. Effect of Seed Priming on Germination Traits

**Figure 2:**
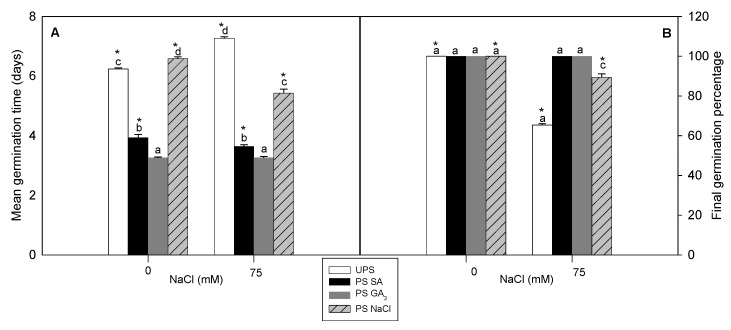
Effects of different seed priming agents on the germination characteristics; Mean of germination time (**A**) and Final germination percentage (**B**), of barley seeds grown in saline medium. UPS, unprimed seed; PS SA, primed seeds with salicylic acid; PS GA_3_, primed seeds with gibberellic acid; and PS NaCl, primed seeds with NaCl. Data are the means of four replicates ± SE. Means followed by different letters significantly differ (*p* ≤ 0.05) as determined using one-way ANOVA. The comparisons between all priming treatments under the different salt stress conditions were statistically analyzed through TWO-WAY ANOVA using SPSS software version 21.0. The asterisks indicate significant differences (*p* ≤ 0.05).

Both hormopriming techniques significantly decreased MGT (mean germination time) irrespective of the salt treatment, although the beneficial effects of seed halopriming were less pronounced (Figure 2A). In non-saline conditions, primed and unprimed seeds had an FGP of 100% (Figure 2B). In saline conditions, unprimed seeds had an FGP (final germination percentage) of 65%, increasing to 89% with NaCl priming and 100% with SA and GA_3_ priming (Figure 2B).

### 3.2. Effect of Seed Priming on Plant Growth and Water Status

**Figure 3:**
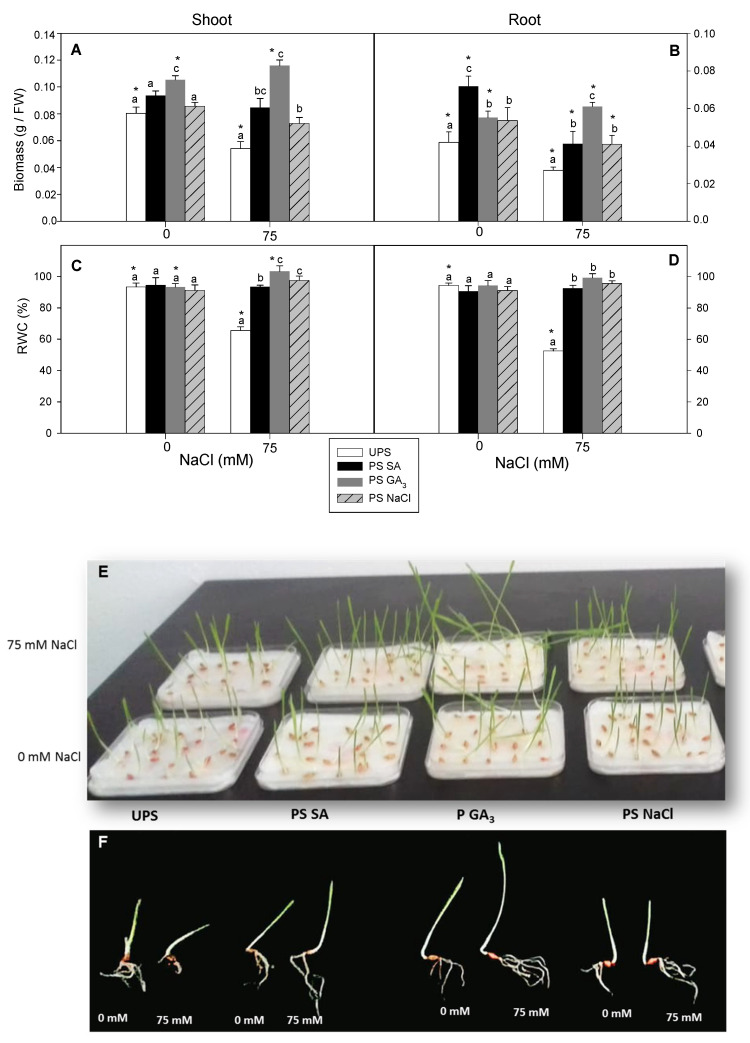
Effects of different seed priming agents on biomass and relative water content (RWC) in shoots (**A**,**C**) and roots (**B**,**D**) of barley seedlings grown under salt stress. UPS, unprimed seed; PS SA, primed seeds with salicylic acid; PS GA_3_, primed seeds with gibberellic acid; and PS NaCl, primed seeds with NaCl. Data are the means of four replicates ± SE. Means followed by different letters significantly differ (*p* ≤ 0.05) as determined using one-way ANOVA. The comparisons between all priming treatments under the different salt stress conditions were statistically analyzed through TWO-WAY ANOVA using SPSS software version 21.0. The asterisks indicate significant differences (*p* ≤ 0.05). (**E**,**F**) Morphological aspect of barley seedlings grown under saline (75 mM NaCl) and non-saline conditions (0 mM NaCl) during 14 days.

In non-saline conditions, only GA_3_ seed priming enhanced shoot growth (Figure 3A). In saline conditions, shoot growth increased the most with GA_3_ seed priming, followed by SA and NaCl. Moreover, unprimed seed treatments exposed to salinity had less shoot growth than primed seed treatments (Figure 3A).

All seed priming treatments increased root weight. In non-saline conditions, seeds primed with SA increased root fresh weight by ~75%, followed by GA_3_, NaCl, and unprimed seeds (Figure 3B). In saline conditions, seeds primed with GA_3_ increased root weight the most, followed by SA, NaCl, and unprimed seeds. Halopriming and hormopriming alleviated the harmful effects of NaCl on plant growth, with shoot and root FWs reaching the control level in seedlings from SA- and NaCl-primed seeds and exceeding the control level in seedlings from GA_3_-primed seeds.

Seedlings from unprimed seeds exposed to salt stress had about 31% lower shoot RWCs than those from primed seeds (Figure 3C). Interestingly, seeds primed with GA_3_ and NaCl had higher shoot RWCs than seeds primed with SA (Figure 3C). Salt stress decreased root RWC by about 45% compared to unstressed seedlings (Figure 3D). All priming treatments significantly increased root RWC under salt stress.

The seedling vigor of unprimed seeds significantly decreased under salt stress compared to the control (Figure 3E,F). All seed priming treatments improved plant vigor under saline conditions, as evidenced by the increased coleoptile length (Figure 3E,F).

### 3.3. Effect of Seed Priming on Chlorophyll Content

**Figure 4:**
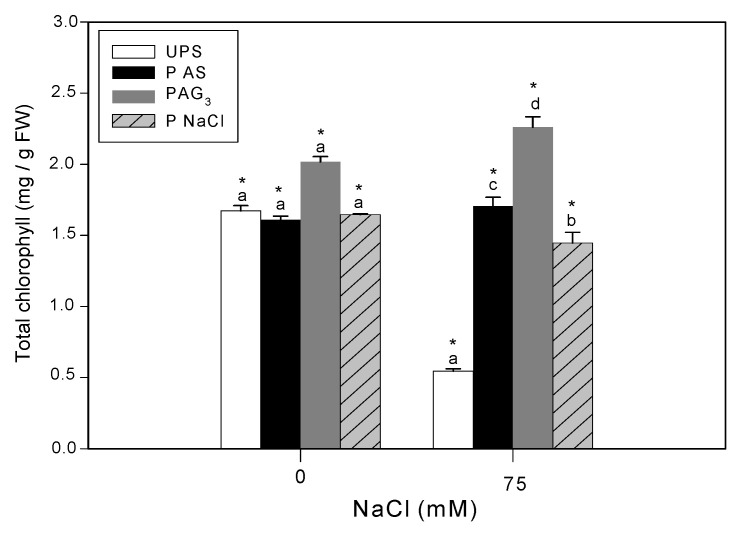
Effects of different seed priming agents on total chlorophyll content in shoots of barley seedlings grown under salt stress. UPS, unprimed seed; PS SA, primed seeds with salicylic acid; PS GA_3_, primed seeds with gibberellic acid; and PS NaCl, primed seeds with NaCl. Data are the means of four replicates ± SE. Means followed by different letters significantly differ (*p* ≤ 0.05) as determined using one-way ANOVA. The comparisons between all priming treatments under the different salt stress conditions were statistically analyzed through TWO-WAY ANOVA using SPSS software version 21.0. The asterisks indicate significant differences (*p* ≤ 0.05).

In seedlings from unprimed seeds, salinity stress decreased chlorophyll by 67% compared to the control (Figure 4). All priming agents alleviated the adverse effects of salt stress on chlorophyll content, increasing 2.7-fold in seeds primed with NaCl, 3-fold in seeds primed with SA, and 4-fold in seedlings primed with GA_3_ compared to unprimed stressed seedlings.

### 3.4. Effect of Seed Priming on Na^+^ and K^+^ Accumulation

**Figure 5:**
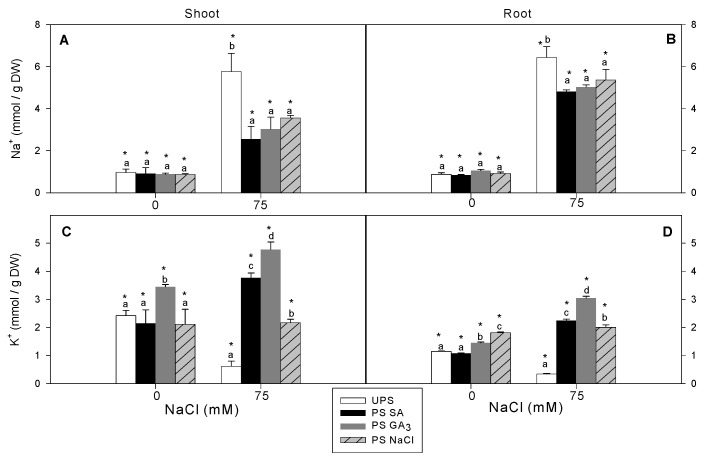
Effects of different seed priming agents on Na^+^ and K^+^ contents in shoots (**A**,**C**) and roots (**B**,**D**) of barley seedlings grown under salt stress. UPS, unprimed seed; PS SA, primed seeds with salicylic acid; PS GA_3_, primed seeds with gibberellic acid; and PS NaCl, primed seeds with NaCl. Data are the means of four replicates ± SE. Means followed by different letters significantly differ (*p* ≤ 0.05) as determined using one-way ANOVA. The comparisons between all priming treatments under the different salt stress conditions were statistically analyzed through TWO-WAY ANOVA SPSS software version 21.0. The asterisks indicate significant differences (*p* ≤ 0.05).

Unprimed salt-stressed seedlings accumulated 5.5–6.5 mmol Na^+^ g^–1^ DW in the shoots and roots, respectively (Figure 5A,B). Seed priming markedly reduced Na^+^ accumulation, particularly in the shoots where it decreased by approximately half. Salt stress decreased K^+^ content by about 75% in the shoots and 71% in the roots (Figure 5C,D). However, seed priming significantly increased K^+^ contents to values equal to or higher than those of the control. Seedlings from GA_3_-primed seeds exposed to salinity stress had the greatest increases in K^+^ contents in the shoots (7.7-fold) and the roots (9.5-fold) compared to salt-treated seedlings from unprimed seeds.

### 3.5. Effect of Seed Priming on H_2_O_2_ and MDA Contents

**Figure 6:**
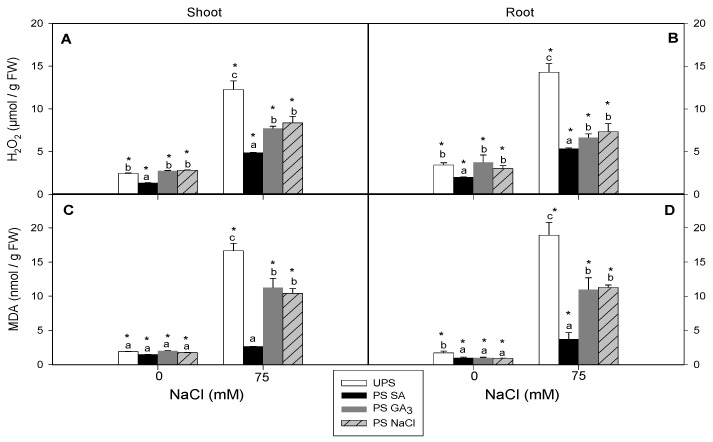
Effects of different seed priming agents on H_2_O_2_ and MDA contents in shoots (**A**,**C**) and roots (**B**,**D**) of barley seedlings grown under salt stress. UPS, unprimed seed; PS SA, primed seeds with salicylic acid; PS GA_3_, primed seeds with gibberellic acid; PS NaCl, primed seeds with NaCl. Data are means of four replicates ± SE. Means followed by different letters significantly differ (*p* ≤ 0.05) as determined using one-way ANOVA. The comparisons between all priming treatments under the different salt stress conditions were statistically analyzed through TWO-WAY ANOVA using SPSS software version 21.0. The asterisks indicate significant differences (*p* ≤ 0.05).

In seedlings from unprimed seeds, salt stress significantly increased H_2_O_2_ and MDA accumulation in shoot and root tissues. However, seedlings from primed seeds had significantly reduced oxidative damage (Figure 6A–D).

Seed priming with SA had a more pronounced effect on H_2_O_2_ and MDA accumulation than on GA_3_ and NaCl under salt stress, reducing shoot and root H_2_O_2_ accumulation by 43 and 65%, respectively, and MDA contents by 80–84%. However, seed priming with GA_3_ resulted in the lowest H_2_O_2_ and MDA accumulation in the shoots and roots among all priming treatments.

### 3.6. Effect of Seed Priming on Total Sugar and Proline Contents

**Figure 7:**
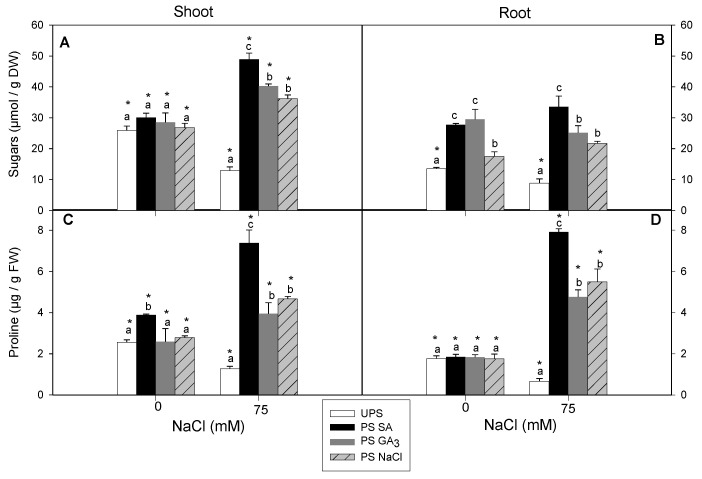
Effects of different seed priming agents on total soluble sugar and proline contents in shoots (**A**,**C**) and roots (**B**,**D**) of barley seedlings grown under salt stress. UPS, unprimed seed; PS SA, primed seeds with salicylic acid; PS GA_3_, primed seeds with gibberellic acid; and PS NaCl, primed seeds with NaCl. Data are means of four replicates ± SE. Means followed by different letters significantly differ (*p* ≤ 0.05) as determined using one-way ANOVA. The comparisons between all priming treatments under the different salt stress conditions were statistically analyzed through TWO-WAY ANOVA using SPSS software version 21.0. The asterisks indicate significant differences (*p* ≤ 0.05).

Salt stress decreased total sugar contents by 48% in the shoots and 25% in the roots of seedlings from unprimed seeds compared to control plants (Figure 7A,B). In non-saline conditions, seed priming increased the total sugar content in the roots but not the shoots. However, in saline conditions, total sugar contents increased in both organs, with the highest levels in seedlings from seeds primed with SA.

For seedlings grown from unprimed seeds, salt stress decreased proline contents by 50% in the shoots and 61% in the roots (Figure 7C,D). In non-saline conditions, the only noticeable change in proline content occurred in the shoots of seedlings from SA-primed seeds (by 42% of increase in comparison with shoots of unprimed seeds). However, in saline conditions, all seed priming treatments increased proline accumulation in the shoots and roots, with the effect of SA priming the most pronounced.

### 3.7. Effect of Seed Pre-Treatment on AsA Accumulation

**Figure 8:**
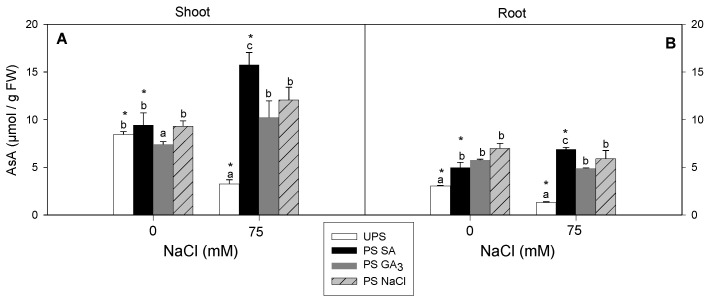
Effects of different seed priming agents on ascorbic acid (AsA) contents in shoots (**A**) and roots (**B**) of barley seedlings grown under salt stress. UPS, unprimed seed; PS SA, primed seeds with salicylic acid; PS GA_3_, primed seeds with gibberellic acid; and PS NaCl, primed seeds with NaCl. Data are means of four replicates ± SE. Means followed by different letters significantly differ (*p* ≤ 0.05) as determined using one-way ANOVA. The comparisons between all priming treatments under the different salt stress conditions were statistically analyzed through TWO-WAY ANOVA using SPSS software version 21.0. The asterisks indicate significant differences (*p* ≤ 0.05).

Salinity stress decreased AsA accumulation by about 61% in the shoots and 56% in the roots (Figure 8A,B). However, seed priming, particularly SA, increased AsA accumulation to levels exceeding the controls.

### 3.8. Effect of Seed Priming on Antioxidant Enzyme Activities (SOD, CAT, and GPX)

**Figure 9:**
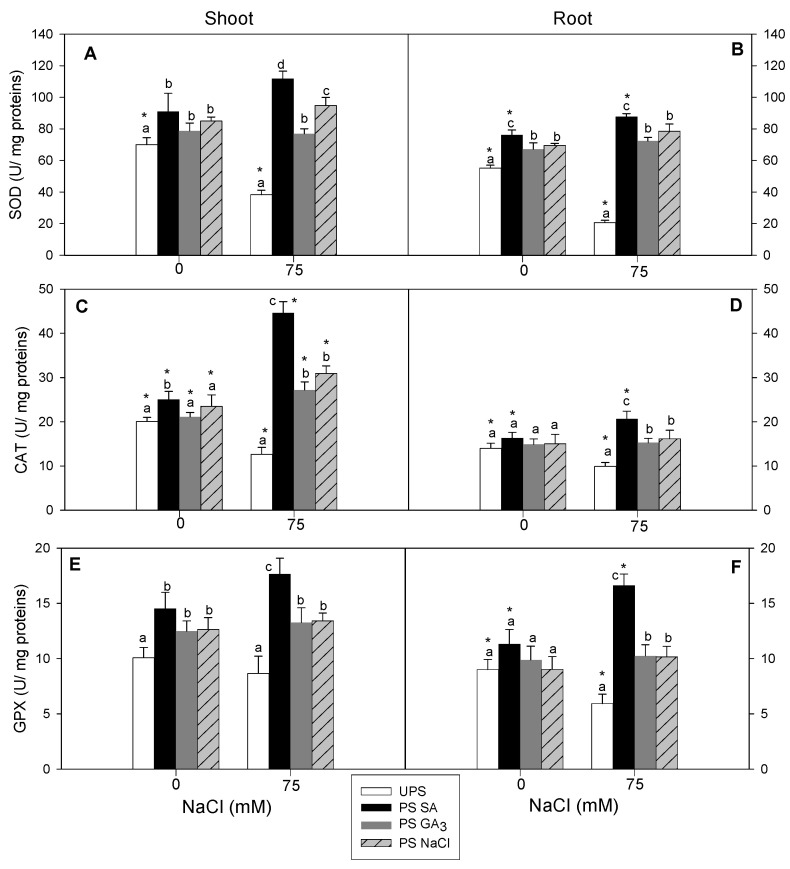
Effects of different seed priming agents on SOD, CAT, and GPX activities in shoots (**A**,**C**,**E**) and roots (**B**,**D**,**F**) of barley seedlings grown under salt stress. UPS, unprimed seed; PS SA, primed seeds with salicylic acid; PS GA_3_, primed seeds with gibberellic acid; and PS NaCl, primed seeds with NaCl. Data are means of four replicates ± SE. Means followed by different letters significantly differ (*p* ≤ 0.05) as determined using one-way ANOVA. The comparisons between all priming treatments under the different salt stress conditions were statistically analyzed through TWO-WAY ANOVA using SPSS software version 21.0. The asterisks indicate significant differences (*p* ≤ 0.05).

Salt stress significantly decreased SOD and CAT activities in the shoots and roots of seedlings from unprimed seeds. Salt stress decreased SOD activity by 45% in the shoots and 63% in the roots (Figure 9A,B). For GPX, the most pronounced decrease was recorded in salt-stressed roots (37%) Seed priming, especially SA, increased SOD activity above the control levels in both organs. Salt stress decreased CAT activity by 37% in the shoots and 29% in the roots (Figure 9C,D). Seed priming alleviated the detrimental effects of salt stress on CAT activity, particularly in the shoots with SA priming. Again, GPX activity increased significantly in salt-stressed roots (46%) derived from SA-primed seeds (Figure 9E,F).

### 3.9. Effect of Seed Priming on Shoot PAL and TAL Activities and Polyphenol Content

**Figure 10:**
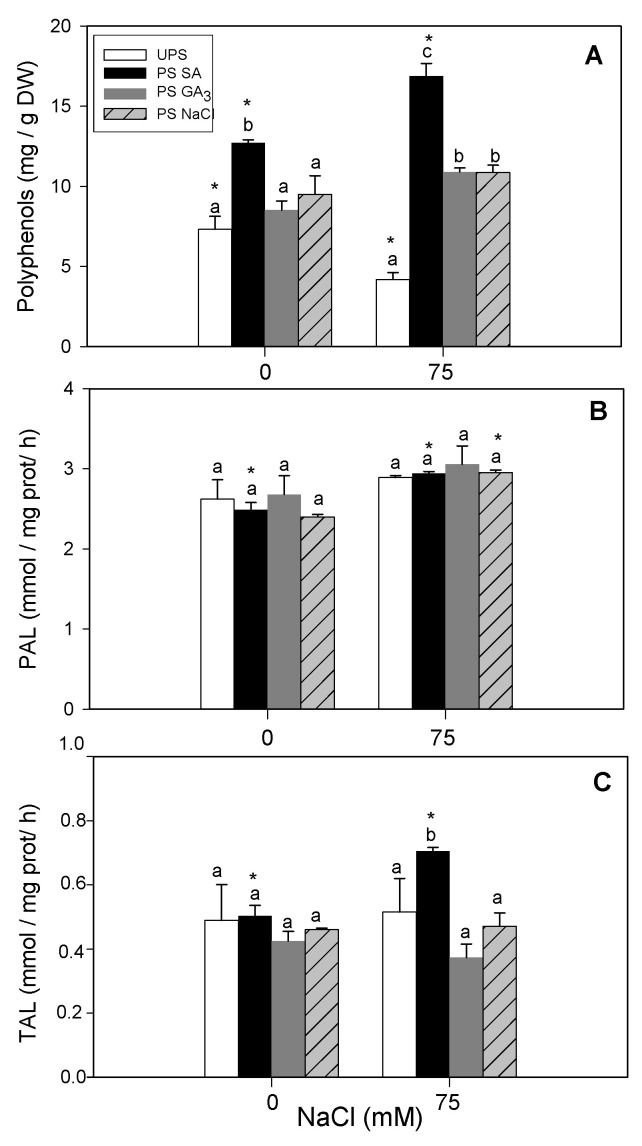
Effects of different seed priming agents on polyphenol content (**A**), PAL (**B**), and TAL (**C**) activities in shoots of barley seedlings grown under salt stress. UPS, unprimed seed; PS SA, primed seeds with salicylic acid; PS GA_3_, primed seeds with gibberellic acid; and PS NaCl, primed seeds with NaCl. Data are means of four replicates ± SE. Means followed by different letters significantly differ (*p* ≤ 0.05) as determined using one-way ANOVA. The comparisons between all priming treatments under the different salt stress conditions were statistically analyzed through TWO-WAY ANOVA using SPSS software version 21.0. The asterisks indicate significant differences (*p* ≤ 0.05).

Salt stress decreased polyphenol contents (Figure 10A) but did not affect PAL and TAL activities (Figure 10B,C). Seed priming with SA increased shoot polyphenol content by 120% in salt-stressed seedlings compared to unprimed seeds (Figure 10A). Seed priming did not affect PAL activity in seedlings under salt stress (Figure 10B). However, seed priming with SA increased TAL activity by 45% under saline conditions (Figure 10C).

## 4. Discussion

Improving salt tolerance in crops has become crucial for using saline lands and increasing productivity. Seed priming is an important technique for enhancing germination performance and seedling growth under saline conditions [4] by controlling osmoregulation processes [56]. In the present study, we investigated the effects of three seed priming agents (NaCl, GA, and SA) on barley germination dynamics and early seedling growth under salt stress. We also evaluated the oxidative stress response, focusing on ROS accumulation and the activation of antioxidant (enzymatic and non-enzymatic) systems.

### 4.1. Growth and Physiological Alterations with GA_3_ and NaCl Seed Priming

The results showed that the seed priming treatments (SA, GA_3_, and NaCl) significantly improved seed vigor and germination performance, as indicated by a decreased mean germination time (MGT) and increased final germination percentage (FGP) under salt stress. Among the priming treatments, GA_3_ seed priming had the most beneficial effect on germination, aligning with the role of GA_3_ in regulating seed germination, as reviewed by Ravindran and Kumar [57]. GA_3_ seed priming can also overcome seed dormancy by stimulating embryo growth and mobilizing reserves in maize plants [58]. In other studies, seed priming with GA_3_ improved rice seedling emergence even under low-temperature stress [59], and halopriming (KNO_3_) or hormopriming (GA_3_) improved wheat and oat seed germination, while hormopriming (IAA or GA_3_) improved barley germination under salt stress [60].

Hormopriming and halopriming alleviated the adverse effects of salt stress on shoot and root growth and water content. Among the priming agents, GA_3_ seed priming had the most significant increase in growth parameters, possibly by increasing cell division and elongation under stressed conditions [61]. Therefore, the improved growth in our study for GA_3_-primed seeds could be attributed to the stimulation of endogenous gibberellin production, as reported by Rodriguez et al. [62]. Saeidi-Sar et al. [63] also observed enhanced growth in plants from GA_3_-primed seeds, which was associated with GA_3_-mediated invertase activity, an enzyme involved in shoot elongation that can lead to hexose accumulation, essential for primary cell wall biosynthesis.

The growth performance of plants under salt stress is closely linked to their ability to regulate Na^+^ accumulation in leaf and root tissues [64]. Our results showed that seed priming, regardless of the agent used, significantly reduced Na^+^ concentrations in the leaves and roots compared to unprimed seeds. At the same time, seed priming increased leaf and root K^+^ contents, particularly from GA_3_-primed seeds. Similarly, Iqbal and Ashraf [65] reported that salt-stressed wheat accumulated less Na^+^ and more K^+^ following seed priming with gibberellin. Mohammed [66] reported that salt-stressed plants from GA_3_-primed seeds decreased Na^+^ and Cl^–^ accumulation, which correlated with increased K^+^ and Ca^2+^ levels, relative to unprimed seeds. These findings indicate that seed priming with GA_3_ enhances Na^+^ exclusion and K^+^ accumulation in plant tissues, which play crucial roles in maintaining ion homeostasis during salt stress. The ameliorative effects of GA_3_ seed priming on germination have been well documented [67], but its role in regulating ion homeostasis under salt stress is not well known. Ahmad et al. [68] proposed that GA_3_ reduced Na^+^ toxicity in salt-stressed Pisum sativum by upregulating Na^+^/H^+^ antiporter genes, leading to the activation of *SOS1* and *NHX1*, which help maintain ion concentrations in the cytosol and enhance salt tolerance. Choudhary et al. [69] suggested that GA3 may interact with the salt overly sensitive pathway through the Ca^2+^ signaling pathway, which mediates plant responses to salt stress. Shukry and El-Bassiouny [70] hypothesized that the reduced Na^+^ accumulation and increased K^+^ ions observed in *Vicia faba* under salt stress were associated with the synergistic effect of gibberellic acid on the activation of salt-responsive proteins, such as osmotin, dehydrin, and ubiquitin, essential for optimal growth. We propose that the GA_3_ present in primed seeds activates early changes that may be associated with the upregulation of Na^+^/K^+^ transporters. The beneficial effect of GA_3_ on K^+^ levels, which is crucial for osmotic adjustment, could be attributed to the activation of various osmoregulatory enzymes in the developing embryo, which are then transported to young seedlings to help them overcome the subsequent osmotic stress [15]. However, further research is needed to explore the interplay between GA_3_ as a priming factor, Na^+^/K^+^ homeostasis, and osmoprotectants during the priming process.

### 4.2. SA Seed Priming Enhances Antioxidant Defense

The disturbances in chlorophyll content, water status, and seedling growth under salt stress could partly be attributed to ROS accumulation. Salt stress typically induces ROS overproduction, including H_2_O_2_, O^2–^, and HO^2–^, which damage cell membranes and structures, resulting in lipid peroxidation (oxidative stress indicator) [71]. We found a significant increase in H_2_O_2_ levels in the shoots and roots of unprimed seeds under saline conditions, accompanied by pronounced lipid peroxidation. In unprimed cauliflower, salt stress decreased membrane stability due to increased ROS production, leading to lipid peroxidation and cell injury, while the opposite profile occurred for primed seeds. In the present study, seedlings from primed seeds grown in saline media substantially decreased H_2_O_2_ and MDA contents in the shoots and roots. Seed priming with SA reduced H_2_O_2_ and MDA accumulation more than GA_3_ and NaCl priming, which may be due in part to the restoration of seed membranes and organelles after priming [15,34]. Hongna et al. [1] also found that SA seed priming significantly decreased MDA and H_2_O_2_ contents in salt-stressed Leymus chinensis plants. These results suggest that ROS, especially H_2_O_2_, act as effective signaling molecules under combined seed priming and salt stress. Moreover, ROS and plant hormones may coexist during abiotic stress, including salinity, such as ROS and GA [72] and ROS and SA [34].

**Figure 11:**
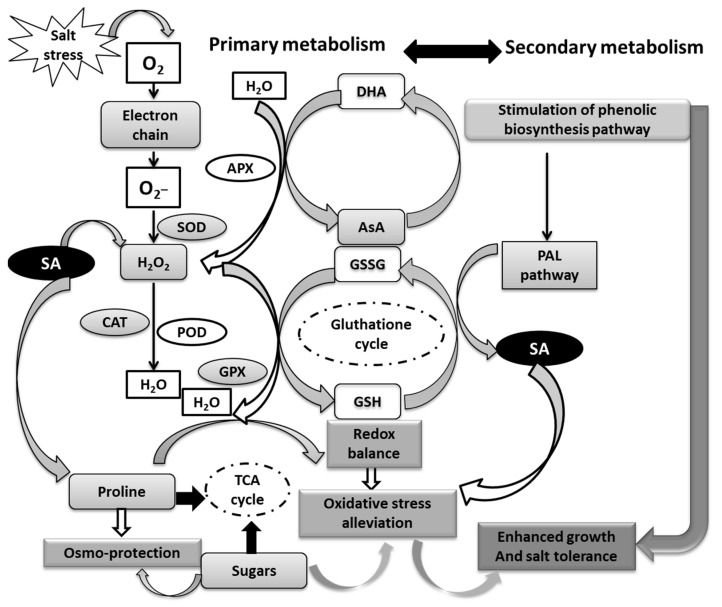
Hypothetical diagram of the cross-talk between SA, ROS, osmolytes, and PAL pathway in seedlings from primed seeds with SA.

Our previous research supports the current findings, showing that priming cauliflower seeds with H_2_O_2_ increased O^2–^, H_2_O_2_, and MDA production while activating the overall antioxidant system, including enzymatic (SOD, CAT, GPX, and APX) and non-enzymatic systems (AsA, GSH, and proline), in H_2_O_2_-primed seeds [15]. Thus, these compounds (O^2–^, H_2_O_2_, and MDA), despite being damaging agents, may also serve as crucial signaling molecules during primed seed germination. ROS has been associated with seed dormancy and germination [73]. In the current study, priming barley seeds with NaCl, GA_3_, or SA alleviated the oxidative damage caused by salinity stress. Notable, barley seedlings from seeds primed with SA had the highest overall antioxidant levels under saline and control conditions. Our results demonstrate that SA seed priming significantly increased shoot and root AsA contents, regardless of NaCl application. This finding is consistent with Wiciarz et al. [74], who suggested that enhanced AsA levels with SA priming can reduce oxidative damage in PSII by detoxifying ROS. This may partly explain the higher AsA levels typically observed in the leaves from SA-primed seeds. Furthermore, this suggests that SA is an effective regulator of the redox state mediated by the ascorbate–glutathione (AsA–GSH) cycle, which plays a key role in H_2_O_2_ detoxification [75]. Several studies have reported that exogenous SA treatment improves the salt stress response in plants such as tomato [76] and maize [77] by increasing AsA accumulation. Our results showed that SA seed priming significantly increased the proline content in salt-stressed barley leaves and roots compared to the unprimed state. Proline provides osmotic adjustment under salinity [78] and functions as a molecular chaperone by scavenging ROS and regulating the cellular redox state [79,80]. The specific increase in shoot and root proline contents under combined salinity and priming suggests that proline accumulation results from the tissue response to the priming agent rather than a reaction to osmotic stress. In lentil, SA seed priming increased proline accumulation by improving γ-glutamyl kinase activity and reducing proline oxidase activity [81]. In *Torreya grandis*, SA seed priming improved the salt stress response through proline synthesis associated with the biosynthesis of stress-protective proteins such as dehydrins [82]. Sharma et al. [35] suggested that increased proline production after SA application is related to the regulation of gene expression, such as *P5CSA* and *P5CSB*, which encode pyrroline-5-carboxylate synthase involved in proline biosynthesis. The positive effect of SA priming on sugar content, which correlated with decreased H_2_O_2_ and MDA levels, suggests that sugar accumulation is activated to counteract the detrimental effects of oxidative stress damage. Sugars may act as ROS scavengers and membrane protectors, with increased sugar accumulation possibly related to the recovery of photosynthetic attributes indirectly linked to redox homeostasis under stressful conditions [83]. To protect themselves from salt-induced stress, plants can decrease salt ion uptake into the cytoplasm, increase osmolyte (organic and inorganic) accumulation, or activate antioxidant systems [84]. In our study, all seedlings originating from primed seeds had higher SOD, CAT, and GPX activities than those from unprimed seeds. However, specific differences related to plant organs and priming agents were observed. Seed priming with SA had the most beneficial effect on the overall dynamics of antioxidant enzymes in the leaves and roots compared to NaCl and GA_3_. These findings are consistent with a study on pistachio seeds treated with SA, which demonstrated improved SOD, CAT, and POX activities in salt-stressed plants, with a more prominent effect in the shoots than the roots [85]. This response was correlated with a decrease in Na^+^ content, which was lowest in salt-stressed leaves from SA-primed seeds. At the same time, this effect was consistent with the most significant decrease in shoot H_2_O_2_ and MDA contents. No significant differences between NaCl and GA_3_ treatments were observed in the shoots or roots in terms of oxidative-stress-related attributes, suggesting that their impact is essential for water uptake, particularly for seedling growth [85]. The present findings support our previous research showing that seed soaking with NaCl or GA_3_ enhanced the growth and water status of cauliflower seedlings under severe salinity stress [15]. Thus, it can be concluded that a strong correlation exists between the priming agent and the plant’s ability to distinguish and use the preferred priming agent to enhance its ability to cope with environmental stressors. Our previous work in barley seedlings showed that priming seeds with silicon (Si) diverted antioxidant systems in the roots, the first site of salt signal detection, to the shoots to maintain redox balance [13]. Based on our findings, it is reasonable to suggest that SA, as a fundamental signal molecule, is specifically recommended for seed priming to repair oxidative damage, particularly in leaves, when seedlings are subjected to subsequent salt stress. Recent studies have highlighted the connections and crosstalk between the SA signaling pathway, redox homeostasis, osmolytes (such as proline and sugars), and antioxidant systems under abiotic stresses, including salinity [86,87]. For example, SA seed priming in peas (*Pisum sativum*) increased antioxidant defense systems and improved the accumulation of osmotic regulators, including soluble sugars and proline [69]. The protective role of SA has been associated with the regulation of ROS and antioxidants [88,89]. In addition, plants have been reported to resist salt-induced oxidative stress by accumulating polyphenolic compounds (secondary metabolites) and activating the PAL pathway [90]. In our study, all seed priming treatments increased polyphenol accumulation when germination occurred under saline conditions compared to the unprimed state, with seeds primed with SA producing the highest total polyphenols. Similarly, PAL and TAL activities increased under saline conditions compared to non-saline conditions. However, no significant differences were observed in PAL activity among priming agents under salt stress, while TAL activity was highest in the shoots from SA-primed seeds. These findings differ from those of Sheteiwy et al. [91], who reported enhanced PAL activity in *Oryza sativa* seedlings primed with SA and subsequently exposed to salt stress. Conversely, wheat seedlings treated with SA and exposed to salinity exhibited increased PAL activity [92]. Another study reported that the increase in total phenolic content in *Artemisia aucheri* primed with SA and subjected to drought stress, during four weeks, was associated with increased PAL and TAL activities [93], with similar observations reported by Dogbo et al. [94]. Based on these findings, the contribution of PAL and TAL may depend on plant species and the type of stress applied (abiotic or biotic stress). Some investigations have proposed that this differential role can be explained by the association between PAL activity and enzymes from dicots and monocots, while TAL activity is more prevalent in monocots [95]. PAL and TAL are key enzymes in the phenylpropanoid pathway. While PAL gene expression and activity have been studied in various plant species, especially under abiotic stresses, TAL activity has not been well studied, particularly in terms of priming and salt stress responses. Our findings clearly showed that the salt tolerance of barley seedlings issued from SA-primed seeds is greatly associated with the crosstalk between SA itself and ROS, osmolytes, and PAL pathways, since SA is an essential signal in plants against salt stress (Figure 11).

## 5. Conclusions

Our study demonstrates the effectiveness of seed priming treatments (halopriming and hormopriming) in improving plant performance and reducing salt-induced damage. Both halopriming and hormopriming techniques enhanced various growth parameters under saline conditions. Seed priming with GA_3_ significantly improved early photosynthesis, growth, and water adjustment, while SA emerged as a powerful signaling molecule for coordinating with other signals in response to combined priming and salt stress. Seed priming with SA enhanced salt tolerance in barley by (i) reducing Na^+^ uptake and thus maintaining Na^+^/K^+^ homeostasis and increasing osmolyte production, (ii) preventing ROS accumulation and thus lipid peroxidation, and (iii) boosting redox signaling and antioxidant defense mechanisms mediated by enzymatic and non-enzymatic antioxidant systems. Thus, when applied early during seed priming, SA can crosstalk with numerous regulatory processes, coordinating their action and execution by plants exposed to salinity. However, the precise mechanisms and interactions involved in the crosstalk between SA and other signaling components during seed priming and the salt stress response remain unclear. Further research is needed to elucidate the intricate network of interactions among these signaling pathways. Understanding these interactions will contribute to the development of more effective strategies for enhancing plant tolerance to salinity and other abiotic stresses.

## Data Availability

Data are available from the corresponding author, Kamel Hessini, upon request.

## References

[1] Hongna C., Leyuan T., Junmei S., Xiaori H., Cheng Xianguo C. (2021). Exogenous salicylic acid signal reveals an osmotic regulatory role in priming the seed germination of *Leymus chinensis* under salt-alkali stress. Environ. Exp. Bot..

[2] Khan A., Anwar Y., Hasan M.M., Iqbal A., Ali M., Alharby H.F., Hakeem K.R., Hasanuzzaman M. (2019). Attenuation of drought stress in brassica seedlings with exogenous application of Ca^2+^ and H_2_O_2_. Planta.

[3] Ibrahim E.A. (2016). Seed priming to alleviate salinity stress in germinating seeds. J. Plant Physiol..

[4] Benidire L., Daoui K., Fatemi Z.A., Achouak W., Bouarab L., Oufdou K. (2015). Effect of salt stress on germination and seedling of *Vicia faba* L. J. Mater. Environ. Sci..

[5] Jisha K.C., Vijayakumari K., Puthur J.T. (2013). Seed priming for abiotic stress tolerance: An overview. Acta Physiol. Plant..

[6] Wani A.S., Ahmad A., Hayat S., Tahir I. (2020). Epibrassinolide and proline alleviate the photosynthetic and yield inhibition under salt stress by acting on antioxidant system in mustard. Plant Physiol. Biochem..

[7] Wojtyla L., Paluch-Lubawa E., Nowicka E.S., Garnczarska M. (2020). Drought stress memory and subsequent drought stress tolerance in plants. Priming-Mediated Stress and Cross-Stress Tolerance in Crop Plants.

[8] Biswas S., Seal P., Majumder B., Biswas A.K. (2023). Efficacy of seed priming strategies for enhancing salinity tolerance in plants: An overview of the progress and achievements. Plant Stress.

[9] Liu X., Quan W., Bartels D. (2022). Stress memory responses and seed priming correlate with drought tolerance in plants: An overview. Planta.

[10] Paparella S., Araujo S.S., Rossi G., Wijayasinghe M., Carbonera D., Balestrazzi A. (2015). Seed priming: State of the art and new perspectives. Plant Cell Rep..

[11] Salam A., Khan A.R., Liu L., Yang S., Azhar W., Ulhassan Z., Gan Y. (2022). Seed priming with zinc oxide nanoparticles downplayed ultrastructural damage and improved photosynthetic apparatus in maize under cobalt stress. J. Hazard. Mater..

[12] Karimi S., Torki Z., Nazok-kar Maher M., Yegane N. (2022). Pre-transplant silicon priming improved drought tolerance and biomass partitioning in young tomato plants. Int. J. Veg. Sci..

[13] Ellouzi H., Rabhi M., Khedher S., Debez A., Abdelly C., Zorrig W. (2023). Silicon seed priming enhances salt tolerance of barley seedlings through early ROS detoxification and stimulation of antioxidant defence. Silicon.

[14] Dai L.Y., Zhu H.D., Yin K.D., Du J.D., Zhang Y.X. (2017). Seed priming mitigates the effects of saline-alkali stress in soybean seedlings. Chil. J. Agric. Res..

[15] Ellouzi H., Oueslati O., Hessini K., Rabhi M., Abdelly C. (2021). Seed-priming with H_2_O_2_ alleviates subsequent salt stress by preventing ROS production and amplifying antioxidant defense in cauliflower seeds and seedlings. Sci. Hortic..

[16] Valivand M., Amooaghaie R., Ahadi A. (2019). Seed priming with H_2_S and Ca^2+^ trigger signal memory that induces cross-adaptation against nickel stress in zucchini seedlings. Plant Physiol. Biochem..

[17] Rajora N., Vats S., Raturi G., Thakral V., Kaur S., Rachappanavar V., Deshmukh R. (2022). Seed priming with melatonin: A promising approach to combat abiotic stress in plants. Plant Stress.

[18] Basit F., Bhat J.A., Ulhassan Z., Noman M., Zhao B., Zhou W., Guan Y. (2022). Seed priming with spermine mitigates chromium stress in rice by modifying the ion homeostasis, cellular ultrastructure and phytohormones Balance. Antioxidants.

[19] Salemi F., Esfahani M.N., Tran L.S.P. (2019). Mechanistic insights into enhanced tolerance of early growth of alfalfa (*Medicago sativa* L.) under low water potential by seed-priming with ascorbic acid or polyethylene glycol solution. Indust. Crops Prod..

[20] Carvalho M.E., Agathokleous E., Nogueira M.L., Brunetto G., Brown P.H., Azevedo R.A. (2023). Neutral-to-positive cadmium effects on germination and seedling vigor, with and without seed priming. J. Hazard. Mater..

[21] Muhei S.H. (2018). Seed priming with phytohormones to improve germination under dormant and abiotic stress conditions. Adv. Crop Sci. Technol..

[22] Sytar O., Kumari P., Yadav S., Brestic M., Rastogi A. (2019). Phytohormone priming: Regulator for heavy metal stress in plants. J. Plant Growth Regul..

[23] Fujita M., Hasanuzzaman M. (2022). Approaches to enhancing antioxidant defense in plants. Antioxidants.

[24] Liu L., Cao X., Zhai Z., Ma S., Tian Y., Cheng J. (2022). Direct evidence of drought stress memory in mulberry from a physiological perspective: Antioxidative, osmotic and phytohormonal regulations. Plant Physiol. Biochem..

[25] Singh V.K., Pandey S., Verma N., Singh M., Pandey J., Prasad S.M. (2022). Cereals and Phytohormones Under Salt Stress. Sustainable Remedies for Abiotic Stress in Cereals.

[26] Oracz K., Karpinski S. (2016). Phytohormones signaling pathways and ROS involvement in seed germination. Front. Plant Sci..

[27] Arora H., Singh R.K., Sharma S., Sharma N., Panchal A., Das T., Prasad A., Prasad M. (2022). DNA methylation dynamics in response to abiotic and pathogen stress in plants. Plant Cell Rep..

[28] Sheteiwy M.S., Ulhassan Z., Qi W., Lu H., AbdElgawad H., Minkina T., Dawood M. (2022). Association of jasmonic acid priming with multiple defense mechanisms in wheat plants under high salt stress. Front. Plant Sci..

[29] Singh P., Singh V., Singh N., Pandurangam V.V., Shahi J.P. (2018). Ameliorating effect of seed priming by salicylic acid on biochemical traits in *Rabi* maize (*Zea mays* L.) genotypes under normal and delayed sowing. J. Pharm. Phytochem..

[30] Hatata M.M., Hala M.T., Dowidar S.M. (2009). Phytohormones effect on growth and certain metabolic aspects of NaCl stressed Barley plant. Egypt. J. Exp. Biol..

[31] Iqbal H., Yaning C., Waqas M., Rehman H.M., Sharee F.M., Iqbal S. (2018). Hydrogen peroxide application improves quinoa performance by affecting physiological and biochemical mechanisms under water-deficit conditions. J. Agro Crop Sci..

[32] Rady M.M., Talaat N.B., Abdelhamid M.T., Shawky B.T., Desoky E.M. (2021). Maize (*Zea mays* L.) grains extract mitigates the deleterious effects of salt stress on common bean (*Phaseolus vulgaris* L.) growth and physiology. J. Hortic. Sci. Biotech..

[33] Al-Harthi M.M., Bafeel S.O., El-Zohri M. (2021). Gibberellic acid and jasmonic acid improve salt tolerance in summer squash by modulating some physiological parameters symptomatic for oxidative stress and mineral nutrition. Plants.

[34] Li Z., Xu J., Gao Y., Wang C., Guo G., Luo Y., Huang Y., Hu W., Sheteiwy M.S., Guan Y. (2017). The synergistic priming effect of exogenous salicylic acid and H_2_O_2_ on chilling tolerance enhancement during maize (*Zea mays* L.) seed germination. Front. Plant Sci..

[35] Sharma A., Shahzad B., Rehman A., Bhardwaj R., Landi M., Zheng B. (2019). Response of phenylpropanoid pathway and the role of polyphenols in plants under abiotic stress. Molecules.

[36] Lone W.A., Majeed N., Yaqoob U., John R. (2022). Exogenous brassinosteroid and jasmonic acid improve drought tolerance in *Brassica rapa* L. genotypes by modulating osmolytes, antioxidants and photosynthetic system. Plant Cell Rep..

[37] Tuna A.L., Kaya C., Higgs D., Murillo-Amador B., Aydemir S., Girgin A.R. (2008). Silicon improves salinity tolerance in wheat plants. Environ. Exp. Bot..

[38] Kaya G. (2022). The efficiency of prechilling and gibberellic acid (GA 3) for breaking thermodormancy in lettuce. J. Seed. Sci..

[39] Ashraf M.Y., Zaib-UN-Nisa N.A., Shani M.Y., Naz A., Azmat M., Ashraf I. (2023). Salicylic acid seed priming improved dry biomass and ionic efficiency of mungbean [*Vigna radiata* (l.) wilczek] under salt stress conditions. Pak. J. Bot..

[40] Hoyos M.E., Zhang S. (2000). Calcium-independent activation of salicylic acid-induced protein kinase and a 40-kilodalton protein kinase by hyperosmotic stress. Plant. Physiol..

[41] Al-Mudaris M. (1998). Notes on various parameters recording the speed of seed germination. Der Tropenlandwirt..

[42] Sairam R.K., Rao K.V., Srivastava G.C. (2002). Differential response of wheat genotypes to longterm salinity stress in relation to oxidative stress, antioxidant activity and osmolyte concentration. Plant Sci..

[43] Lichtenthaler H.K. (1987). Chlorophylls and carotenoids: Pigments of photosynthetic biomembranes. Methods in Enzymology.

[44] Yemm E.W., Willis A. (1954). The estimation of carbohydrates in plant extracts by anthrone. Biochem. J..

[45] Zorrig W., Cornu J.Y., Maisonneuve B., Rouached A., Sarrobert C., Shahzad Z., Berthomieu P. (2019). Genetic analysis of cadmium accumulation in lettuce (*Lactuca sativa*). Plant Physiol. Biochem..

[46] Sergiev I., Alexieva V., Karnov E. (1997). Effect of spermine, atrazine and combination between them on some endogenous protective systems and stress markers in plants. Compt. Rend. Acad. Bulg. Sci..

[47] Hodge D.M., DeLong J.M., Forney C.F., Robert K.P. (1999). Improving the thiobarbituric acid reactive-substances assay for estimating lipid peroxidation in plant tissues containing anthocyanin and other interfering compounds. Planta.

[48] Bates L.S., Wadern R.P., Teare I.D. (1973). Rapid determination of free proline for water-stress studies. Plant Soil.

[49] Kampfenkel K., Van Montagu M., Inze D. (1995). Extraction and determination of ascorbate and dehydroascorbate from plant tissue. Anal. Biochem..

[50] Mau J.L., Chao G.R., Wu K.T. (2001). Antioxidantproperties of methanolic extracts from several ear mushrooms. J. Agric. Food Chem..

[51] Bradford M.M. (1976). A rapid and sensitive method for the quantitation of microgram quantities of protein utilizing the principle of protein-dye binding. Anal. Biochem..

[52] Scebba F., Sebastiani L., Vitagliano C. (1999). Protective enzymes against activated oxygen species in wheat (*Triticumaestivum* L.) seedlings: Responses to cold acclimation. J. Plant Physiol..

[53] Lück H., Bergmeyer H.U. (1965). Catalase. Methods of Enzymatic Analysis.

[54] Fielding J.L., Hall J.L. (1978). A Biochemical and Cytochemical Study of Peroxidase Activity in Roots of *Pisumsativum*: I. a comparison of dab-peroxidase and guaiacol-peroxidase with particular emphasis on the properties of cell wall activity. J. Exp. Bot..

[55] Berner M., Krug D., Bihlmaier C., Vente A., Muller R., Bechthold A. (2006). Genes and enzymes involved in caffeic acid biosynthesis in the actinomycete *Saccharothrix espanaensis*. J. Bacteriol..

[56] Rhaman M.S., Imran S., Rauf F., Khatun M., Baskin C.C., Murata Y., Hasanuzzaman M. (2021). Seed priming with phytohormones: An effective approach for the mitigation of abiotic stress. Plants.

[57] Kumar Y., Sharanagat V.S., Singh L., Mani S. (2020). Effect of germination and roasting on the proximate composition, total phenolics, and functional properties of black chickpea (*Cicer arietinum*). Legume Sci..

[58] Pallaoro D.S., Camilli E.S., Guimaraes S.C., Albuquerque M.C.F. (2016). Methods of priming maize seed. J. Seed. Sci..

[59] Chen X., Zhang R., Xing Y., Jiang B., Li B., Xu X., Zhou Y. (2021). The efficacy of different seed priming agents for promoting sorghum germination under salt stress. PLoS ONE.

[60] Kanjevac M., Bojović B., Ćirić A., Stanković M., Jakovljević D. (2022). Seed priming improves biochemical and physiological performance of wheat seedlings under low-temperature conditions. Agriculture.

[61] Nimir N.E.A., Lu S., Zhou G., Guo W., Ma B., Wang Y. (2015). Comparative effects of gibberellic acid, kinetin and salicylic acid on emergence, seedling growth and the antioxidant defence system of sweet sorghum (*Sorghum bicolor*) under salinity and temperature stresses. Crop Pasture Sci..

[62] Rodríguez A., Stella A., Storni M., Zulpa G., Zaccaro M.C. (2006). Effects of cyanobacterial extracellular products and gibberellic acid on salinity tolerance in *Oryza sativa* L. Aquat. Biosyst..

[63] Saeidi-Sar S., Khavari-Nejad R.A., Fahimi H., Ghorbanli M., Majd A. (2007). Interactive effects of gibberellin A3 and ascorbic acid on lipid peroxidation and antioxidant enzyme activities in glycine max seedlings under nickel. Russ. J. Plant Physiol..

[64] Zhao S., Zhang Q., Liu M., Zhou H., Ma C., Wang P. (2021). Regulation of plant responses to salt stress. Inter. J. Mol. Sci..

[65] Iqbal M., Ashraf M. (2016). Gibberellic acid mediated induction of salt tolerance in wheat plants: Growth, ionic partitioning, photosynthesis, yield and hormonal homeostasis. Environ. Exp. Bot..

[66] Mohammed A.H.M.A. (2007). Physiological aspects of mungbean plant (*Vigna radiata* L. Wilczek) in response to salt stress and gibberellic acid treatment. Res. J. Agric. Biol. Sci..

[67] Khan M.M., Usman M., Waseem R., Ali M.A. (2002). Role of gibberellic acid (ga~ 3) on citrus seed germination and study of some morphological characteristics. Pak. J. Agric. Sci..

[68] Ahmad R., Ali S., Rizwan M., Dawood M., Farid M., Hussain A., Leonard Wijaya L., Nasser M.N., Ahmad P. (2020). Hydrogen sulfide alleviates chromium stress on cauliflower by restricting its uptake and enhancing antioxidative system. Physiol. Plant..

[69] Choudhary K.K., Singh S., Agrawal M., Agrawal S.B., Aftab T., Yusuf M. (2021). Role of jasmonic and salicylic acid signaling in plants under UV-B stress. Jasmonates and Salicylates Signaling in Plants.

[70] Shukry W.M., El-Bassiouny H.M.S. (2002). Gibberellic acid effects on protein pattern, hydrolytic enzyme activities and ionic uptake during germination of *Vicia faba* in sea water. Acta Bot. Hungar..

[71] Gill S.S., Tuteja N. (2010). Reactive oxygen species and antioxidant machinery in abiotic stress tolerance in crop plants. Plant Physiol. Biochem..

[72] Colebrook E.H., Thomas S.G., Phillips A.L., Hedden P. (2014). The role of gibberellin signalling in plant responses to abiotic stress. J. Exp. Biol..

[73] Farooq M.A., Zhang X., Zafar M.M., Ma W., Zhao J. (2021). Roles of reactive oxygen species and mitochondria in seed germination. Front. Plant Sci..

[74] Wiciarz M., Niewiadomska E., Kruk J. (2018). Effects of salt stress on low molecular antioxidants and redox state of plastoquinone and P700 in *Arabidopsis thaliana* (glycophyte) and *Eutrema salsugineum* (halophyte). Photosynthetica.

[75] Yan Z., Ming D.I.A.O., Cui J.X., Chen X.J., Wen Z.L., Zhang J.W., Liu H.Y. (2018). Exogenous GSH protects tomatoes against salt stress by modulating photosystem II efficiency, absorbed light allocation and H_2_O_2_-scavenging system in chloroplasts. J. Integ. Agric..

[76] Zhu Z., Chen Y., Shi G., Zhang X. (2017). Selenium delays tomato fruit ripening by inhibiting ethylene biosynthesis and enhancing the antioxidant defense system. Food Chem..

[77] Yan J., Tan Y., Xu L., Lv Y., Wang F., Shan W., Xu D. (2023). Effect of exogenous salicylic acid on salt tolerance of *Hosta ensata*. Eur. J. Hortic. Sci..

[78] Farhangi-Abriz S., Ghassemi-Golezani K. (2018). How can salicylic acid and jasmonic acid mitigate salt toxicity in soybean plants?. Ecotoxicol. Environ. Saf..

[79] Szabados L., Savouré S. (2010). Proline: A multifunctional amino acid. Trends Plant Sci..

[80] Ellouzi H., Sghayar S., Abdelly C. (2017). H_2_O_2_ seed priming improves tolerance to salinity; drought and their combined effect more than mannitol in *Cakile maritima* when compared to *Eutrema salsugineum*. J. Plant Physiol..

[81] Misra N., Saxena P. (2009). Effect of salicylic acid on proline metabolism in lentil grown under salinity stress. Plant Sci..

[82] Li T., Hu Y., Du X., Tang H., Shen C., Wu J. (2014). Salicylic acid alleviates the adverse effects of salt stress in *Torreya grandis* cv. *Merrillii* seedlings by activating photosynthesis and enhancing antioxidant systems. PLoS ONE.

[83] Nazar R., Iqbal N., Syeed S., Khan N.A. (2015). Salicylic acid alleviates decreases in photosynthesis under salt stress by enhancing nitrogen and sulfur assimilation and antioxidant metabolism differentially in two mungbean cultivars. J. Plant Physiol..

[84] Talaat N.B., Abdel Wahab M., Alaa M., Hanafy A. (2023). Co-application of salicylic acid and spermine alleviates salt stress toxicity in wheat: Growth, nutrient acquisition, osmolytes accumulation, and antioxidant response. Acta Physiol. Plant.

[85] Jannesar M., Seyedi M.S., Niknam V., Khorzoghi E.G., Hassan Ebrahimzadeh H. (2022). Salicylic acid, as a positive regulator of isochorismate synthase, reduces the negative effect of salt stress on *Pistacia vera* L. by increasing photosynthetic pigments and inducing antioxidant activity. J. Plant Reg..

[86] Khan M.I.R., Poor P., Janda T. (2022). Salicylic acid: A versatile signaling molecule in plants. J. Plant Growth Regul..

[87] Khalid M.F., Saleem M.S., Zakir I., Khan R.I., Sohail M., Ejaz S., Hussain S. (2023). Salicylic acid induced abiotic stress tolerance in plants. Plant Stress Mitigators.

[88] Al Mahmud J., Bhuyan M.B., Anee T.I., Nahar K., Fujita M., Hasanuzzaman M. (2019). Reactive oxygen species metabolism and antioxidant defense in plants under metal/metalloid stress. Plant Abiotic Stress Tolerance: Agronomic, Molecular and Biotechnological Approaches.

[89] Alamri S.A.D., Siddiqui M.H., Al-Khaishany M.Y., Ali H.M., Al-Amri A., AlRabiah H.K. (2018). Exogenous application of salicylic acid improves tolerance of wheat plants to lead stress. Adv. Agric. Sci..

[90] Yang C., Hu L.Y., Ali B., Islam F., Bai Q.J., Yun X.P., Zhou W.J. (2016). Seed treatment with salicylic acid invokes defence mechanism of Helianthus annuus against Orobanche cumana. Ann. Appl. Biol..

[91] Sheteiwy M.S., An J., Yin M., Jia X., Guan Y., He F., Hu J. (2019). Cold plasma treatment and exogenous salicylic acid priming enhances salinity tolerance of *Oryza sativa* seedlings. Protoplasma.

[92] Saleh A.M., Madany M.M., González L. (2015). The effect of coumarin application on early growth and some physiological parameters in faba bean (*Vicia faba* L.). J. Plant Growth Regul..

[93] Abbaspour J., Ehsanpour A. (2016). The impact of salicylic acid on some physiological responses of *Artemisia aucheri* Boiss. under in vitro drought stress. Acta Agric. Slov..

[94] Dogbo D.O., Gogbeu S.J., N’zue B., Ya K.A., Zohouri G.P., Mamyrbekova-Bekro J.A., Bekro Y.A. (2012). Comparative activities of phenylalanine ammonia-lyase and tyrosine ammonia-lyase and phenolic compounds accumulated in cassava elicited cell. Afr. Crop Sci. J..

[95] Khan W., Prithiviraj B., Smith D.L. (2003). Chitosan and chitin oligomers increase phenylalanine ammonia-lyase and tyrosine ammonia-lyase activities in soybean leaves. J. Plant Physiol..

